# Resistance Analyses of Lenacapavir, Emtricitabine/Tenofovir Alafenamide and Emtricitabine/Tenofovir Disoproxil Fumarate in the PURPOSE 1 and 2 Studies

**DOI:** 10.1093/infdis/jiaf533

**Published:** 2025-10-24

**Authors:** Stephanie Cox, Kristen Andreatta, Matthew R Hendricks, Jiani Li, Alexander Kintu, Lillian B Brown, Christoph C Carter, Christian Callebaut

**Affiliations:** Gilead Sciences, Inc., Foster City, California, USA; Gilead Sciences, Inc., Foster City, California, USA; Gilead Sciences, Inc., Foster City, California, USA; Gilead Sciences, Inc., Foster City, California, USA; Gilead Sciences, Inc., Foster City, California, USA; Gilead Sciences, Inc., Foster City, California, USA; Gilead Sciences, Inc., Foster City, California, USA; Gilead Sciences, Inc., Foster City, California, USA

**Keywords:** HIV, capsid, lenacapavir, resistance, PrEP

## Abstract

**Background:**

Lenacapavir (LEN) is an HIV-1 capsid inhibitor being evaluated for pre-exposure prophylaxis (PrEP). The PURPOSE trials assessed the efficacy of LEN and emtricitabine/tenofovir disoproxil fumarate (F/TDF) in cisgender women (PURPOSE 1; P1) and in cisgender men, transgender women, transgender men and gender non-binary persons (PURPOSE 2; P2). Emtricitabine/tenofovir alafenamide (F/TAF) was also assessed in P1. Both studies demonstrated the superiority of LEN to F/TDF. We describe resistance analyses from P1 and P2, which provide the first data regarding emergent drug resistance in the context of LEN for PrEP.

**Methods:**

HIV testing was performed at screening, baseline, and every study visit. Participants who acquired HIV-1 with a viral load of ≥200 copies/mL were evaluated for resistance by genotyping of the HIV-1 capsid, protease, reverse transcriptase and integrase genes at HIV diagnosis. Adherence in the F/TDF and F/TAF groups was measured by dried blood spot.

**Results:**

Resistance to LEN was detected in 0 of 2134 participants (P1) and 2 of 2179 participants (P2); both developed N74D. Four participants in each study receiving LEN were found to have unrecognized HIV-1 at baseline; 4/8 participants developed N74D. In P1, 2/37 participants analyzed for resistance in the F/TAF group had M184I ± K65R. In the F/TDF groups, M184M/I/V was detected in 1/16 participants (P1) and 1/9 participants (P2).

**Conclusions:**

Acquisition of HIV while receiving LEN and resistance to LEN in the context of unrecognized HIV was rare but associated with the emergence of the N74D LEN resistance-associated substitution in this population.

## Teaser

The resistance analyses in the HIV pre-exposure prophylaxis PURPOSE 1 and 2 studies are described through the primary endpoint. Overall resistance was rare although resistance to LEN was associated with the emergence of the N74D resistance-associated substitution.

Lenacapavir (LEN) is an inhibitor of human immunodeficiency virus type 1 (HIV-1) capsid (CA) function that is approved for HIV-1 treatment by the Food and Drug Administration and European Medicines Agency and recently approved for pre-exposure prophylaxis (PrEP). Other available PrEP options include oral, emtricitabine/tenofovir disoproxil fumarate (F/TDF) and emtricitabine/tenofovir alafenamide (F/TAF), which are highly effective when taken as directed [1–5]. However, several challenges have been identified as barriers to adherence and persistence, including stigma, side effects, low risk perception, and the requirement for daily dosing [6]. LEN, as a twice-yearly injectable agent with picomolar potency targeting multiple stages of the HIV replication cycle, has demonstrated high efficacy, safety and tolerability for PrEP [7–9].

Resistance emergence on PrEP is a concern because often PrEP agents are also recommended for first-line HIV-1 therapy. Resistance surveillance during clinical trials provides an indication of what resistance may emerge when the agent is approved for use. LEN is unique since it has no known cross-resistance to any currently approved antiretroviral classes, but further understanding of how resistance may emerge when it is used as PrEP will help inform the overall resistance profile [10]. In clinical trials of treatment-naïve or heavily treatment-experienced (HTE) people with HIV-1 on a failing antiretroviral regimen, treatment-emergent resistance to LEN consisted of three general patterns of CA substitutions: Q67H ± K70R, M66I ± other CA substitutions (Q67H, K70R/S, N74D, A105T, T107C/A) and K70H/N + other rare CA substitutions (Q67K, N74K) [11–16]. These substitutions, alone or in combination, were associated with 5- to >869-fold reduced susceptibility to LEN, and mostly occurred in participants with functional monotherapy due to non-adherence to the other components of their optimized background antiretroviral regimen (OBR), or in those with no other fully active agents in their antiretroviral regimen caused by pre-existing resistance [17].

The Phase 3 PURPOSE 1 (P1; NCT04994509) and PURPOSE 2 (P2; NCT04925752) studies compared the efficacy of LEN in preventing HIV-1 acquisition relative to the background HIV (bHIV) incidence (primary comparator) and to the efficacy of F/TDF (secondary comparator). Twice-yearly subcutaneous (SC) LEN was highly efficacious in preventing HIV-1 acquisition in diverse groups of people who would benefit from PrEP [8, 9]. In the P1 primary analysis, LEN demonstrated 100% efficacy in reducing HIV-1 incidence versus bHIV incidence and F/TDF [8, 9]. In the P2 primary analysis, LEN demonstrated 96% efficacy in reducing HIV-1 incidence versus bHIV incidence and 89% efficacy in reducing HIV-1 incidence versus F/TDF [9]. Additionally, in P1, F/TAF was also evaluated but was not shown to reduce HIV incidence compared with bHIV incidence due to high rates of non-adherence.

We describe the resistance profiles of participants who acquired HIV in the P1 and P2 trials, providing the first data on resistance emergence when LEN is used as PrEP.

## METHODS

P1 evaluated the safety and efficacy of twice-yearly SC LEN and daily oral F/TAF for PrEP in cisgender adolescent girls and young women who have sex with male partners and are 16–25 years of age [8]. P2 evaluated the safety and efficacy of twice-yearly SC LEN for PrEP in cisgender gay, bisexual and other men who have sex with men, transgender women, transgender men and gender non-binary people who have condomless receptive anal sex with partners assigned male at birth and are ≥16 years of age [9]. All participants provided written informed consent.

Study designs for P1 and P2 have been reported previously (Supplementary Figure 1) [8, 9]. Briefly, participants who tested negative for HIV during the incidence phase were randomized to receive SC LEN, oral F/TDF, or oral F/TAF (P1 only) with the alternative SC or oral placebo. The efficacy analysis population (EAP) included enrolled participants who received at least one dose of study drug and were not diagnosed with HIV-1 by Day 1.

**Figure 1.**
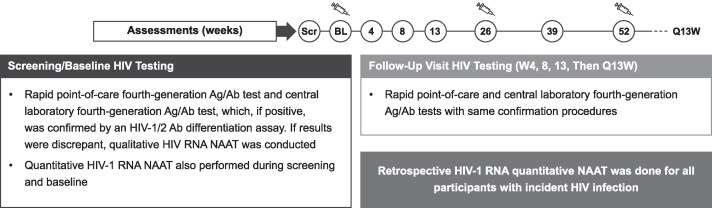
HIV testing during the randomized blinded phase. Syringe symbols indicate timing of LEN injections in the PURPOSE trials. Ab, antibody; Ag, antigen; BL, baseline; LEN, lenacapavir; NAAT, nucleic acid amplification testing; Q13W, every 13 weeks; Scr, screening; W, weeks.

HIV qualitative and quantitative diagnostic testing was performed at screening and every study visit in the randomized blinded phase, with confirmatory testing outlined in Figure 1 [8, 9]. Retrospective HIV-1 RNA quantitative nucleic acid amplification testing (NAAT) was performed using archived samples for incident HIV-1 cases to determine the earliest date with evidence of HIV-1 acquisition.

HIV-1 single-copy assay (SCA) testing was performed retrospectively on the Hologic Panther platform using a multiple replicate strategy (Accelevir Diagnostics, Baltimore, Maryland) on samples from EAP participants in the LEN group who acquired HIV-1 [18, 19].

Upon HIV-1 diagnosis, plasma samples were collected for HIV-1 RNA quantification and resistance analyses. Samples with HIV-1 RNA ≥200 copies/mL were analyzed for genotypic resistance in the HIV-1 CA, protease (PR), reverse transcriptase (RT), and integrase coding regions using the GenoSure® GAG PRO and GenoSure PRIme® assays (Monogram Biosciences, South San Francisco, California), which are validated tests run in a Clinical Laboratory Improvement Amendments–certified laboratory. Genotypic CA resistance testing at Monogram was conducted using a next-generation sequencing platform (MiSeq; Illumina), with reporting of mutants present in ≥10% of sequences obtained for a given sample. The mutant detection cutoff was based on reproducibility and coverage data obtained during the proprietary assay validation process. Participants who received study drug at Day 1 but were later found to have acquired HIV-1 before study initiation were also analyzed for resistance. The list of major primary resistance mutations evaluated was adapted from the most recent International Antiviral Society—USA list with some modifications [20] (Supplementary Table 1).

**Figure 2.**
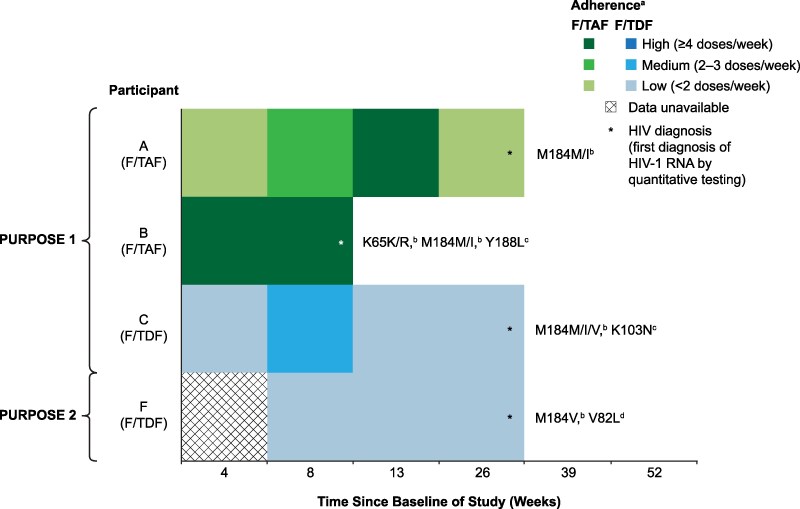
Longitudinal adherence patterns of PURPOSE 1 and PURPOSE 2 participants on oral PrEP with resistance mutations. Each row represents a single participant. Each box represents DBS-based adherence for the corresponding time period. ^a^By TFV-DP DBS levels. For F/TAF, adherence was categorized as the following: low, <450 fmol/punch (dosing <2 days/week); medium, ≥450 to <950 fmol/punch (dosing 2–3 days/week); high, ≥900 fmol/punch (dosing ≥4 days/week). For F/TDF, adherence was categorized as the following: low, <350 fmol/punch (dosing <2 days/week); medium, ≥350 to <700 fmol/punch (dosing 2–3 days/week); high, ≥700 fmol/punch (dosing ≥4 days/week). ^b^NRTI resistance mutation. ^c^NNRTI resistance mutation. ^d^PI resistance mutation. DBS, dried blood spot; F/TAF, emtricitabine/tenofovir alafenamide; F/TDF, emtricitabine/tenofovir disoproxil fumarate; NNRTI, non-nucleoside reverse transcriptase inhibitor; NRTI, nucleoside reverse transcriptase inhibitor; PI, protease inhibitor; TFV-DP, tenofovir diphosphate.

Adherence to daily oral PrEP over the preceding 8–12 weeks was calculated by concentration of tenofovir (TFV) diphosphate in dried blood spot (DBS) testing, as previously described [21, 22]. Details on how adherence was categorized are described in Figure 2.

To evaluate the polymorphic frequencies of CA resistance substitutions in relevant HIV-1 subtypes, 36 875 gag population sequences were downloaded from the HIV Los Alamos National Laboratory (LANL; 12 September 2024 build; LANL problematic sequences excluded) [23]. To avoid redundancy, only one sequence per subject identifier was retained (clonal sequences removed), resulting in 12 075 sequences. The final dataset included *n* = 2946 subtype A1, *n* = 8170 subtype C, *n* = 931 subtype D and *n* = 28 subtype AD sequences. All sequences were aligned to the HIV HXB2 reference sequence using MAFFT [24]. Ambiguous amino acids were resolved based on codon composition to ensure accuracy at resistance mutation sites.

## RESULTS

### PURPOSE 1

**Figure 3.**
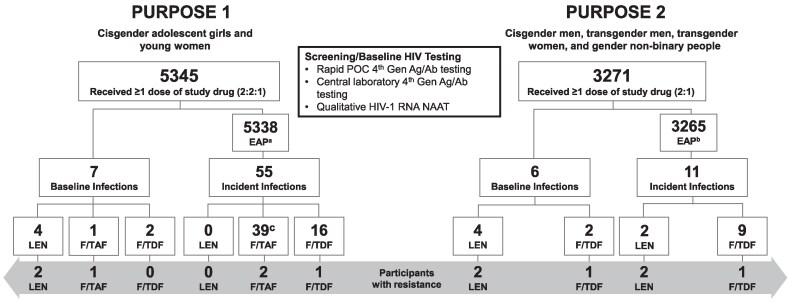
Resistance Testing and Emergent Resistance for Participants Who Acquired HIV in the PURPOSE Trials. ^a^August 2021–September 2023; ^b^June 2021–December 2023; ^c^Two participants were not included in the RAP due to VL <200 copies/mL or withdrawn consent. Ab, antibody; Ag, antigen, EAP, efficacy analysis population; F/TAF, emtricitabine/tenofovir alafenamide; F/TDF, emtricitabine/tenofovir disoproxil fumarate; Gen, generation; LEN, lenacapavir; NAAT, nucleic acid amplification test; POC, point-of-care; RAP, resistance analysis population; VL, viral load.

Of the 5338 participants in the P1 EAP, 55 acquired HIV-1 after Day 1 (at primary analysis; Table 1) [8]. In the LEN group, no participant acquired HIV-1 during the study (Figure 3).

**Table 1.** Study Disposition and Resistance Testing

	LEN	F/TAF	F/TDF	Total
PURPOSE 1				
Efficacy Analysis Population	2134	2136	1068	5338
Acquired HIV	0	39	16	55
Tested for Resistance	0	37^a^	16	53
Resistance Present	0	2	1	3
PURPOSE 2				
Efficacy Analysis Population	2179	N/A	1086	3265
Acquired HIV	2	N/A	9	11
Tested for Resistance	2	N/A	9	11
Resistance Present	2	N/A	1	3

Abbreviations: F/TAF, emtricitabine/tenofovir alafenamide; F/TDF, emtricitabine/tenofovir disoproxil fumarate; LEN, lenacapavir; N/A, not available.

^a^Two participants who acquired HIV-1 were not tested for resistance due to consent being withdrawn prior to testing or viral load <200 copies/mL.

Among the 39 participants in the F/TAF group who acquired HIV-1, 37 were analyzed for resistance, with RT genotypic data available for 36. Resistance-associated substitutions were detected in two participants (Table 2). Participant A harbored the substitution M184M/I in RT at HIV-1 diagnosis (Week 26). Adherence was low at Week 4, improved by Week 13, then declined near the time of HIV-1 acquisition (Figure 2; Supplementary Table 2). The participant declined follow-up care; virologic suppression could not be confirmed although the final viral load was <200 copies/mL (Supplementary Table 3). Participant B had both M184M/I and K65K/R in RT and the non-nucleoside reverse transcriptase inhibitor (NNRTI) substitution Y188L. Adherence remained high through Week 8, when HIV-1 was detected (Figure 2; Supplementary Table 2). Virologic suppression (HIV-1 RNA <50 copies/mL) was achieved on dolutegravir (DTG)/lamivudine (3TC)/TDF within 9 weeks of HIV-1 acquisition (Supplementary Table 3).

**Table 2.** Participants Who Developed Resistance During the Study in PURPOSE 1 and 2

Study	Participant	Group	HIV Subtype	Timepoint of Resistance Testing	Viral Load, Copies/mL	LEN-R	NRTI-R^a^	NNRTI-R	PI-R
PURPOSE 1	A	F/TAF	C	Week 26	4500	–	M184M/I	–	–
	B	F/TAF	C	Week 8	6890	–	K65K/R, M184M/I	Y188L	–
		…	…	Week 13	1510	–	M184I	Y188L	–
	C	F/TDF	C	Week 26	38 200	–	–	K103N	–
		…	…	Week 39	108 000	–	M184M/V/I	K103N	–
PURPOSE 2	D	LEN	C	Week 13	699 000	N74D	–	K103N	–
	E	LEN	C	Week 26	14 100	N74D	–	–	–
	F	F/TDF	C	Week 39	99 700	–	M184V	–	V82L

Abbreviations: –, no resistance mutations detected; F/TAF, emtricitabine/tenofovir alafenamide; F/TDF, emtricitabine/tenofovir disoproxil fumarate; LEN, lenacapavir; NNRTI, non-nucleoside reverse transcriptase inhibitor; NRTI, nucleoside reverse transcriptase inhibitor; PI, protease inhibitor; -R, resistance.

^a^F/TAF and F/TDF are NRTIs.

In the F/TDF group, 16 participants acquired HIV-1; resistance analysis was conducted for all 15 participants who had RT genotypic data available. Participant C had the M184M/V/I mutation in RT at Week 39 when HIV-1 acquisition was detected by the central laboratory (Table 2). However, reflex NAAT of the previous visit demonstrated acquisition of HIV-1 at Week 26. Week 26 adherence was low (Figure 2; Supplementary Table 2). Participant C achieved virologic suppression on DTG/3TC/TDF within 41 weeks of HIV-1 acquisition (Supplementary Table 3).

Additionally, seven P1 participants had unrecognized HIV-1 at baseline (Figure 3). In these cases, Day 1 rapid fourth-generation HIV-1/2 antigen/antibody (Ag/Ab) testing was negative and study drug was started. Results of central laboratory testing collected at Day 1 demonstrated HIV-1 acquisition. All seven received study drug (LEN [*n* = 4], F/TAF [*n* = 1], F/TDF [*n* = 2]) and were analyzed for resistance; none had resistance substitutions detected at Day 1 (Table 3). Follow-up data indicated that two of four participants who received LEN developed the N74D substitution in CA (Participants G and H; Table 3). Participant G had a high HIV-1 RNA viral load (105 000 000 copies/mL) at Day 1; the N74N/D substitution emerged by Day 9. DTG/3TC/TDF was initiated at Day 141, with viral suppression (<200 copies/mL) by Day 269. Participant H had a viral load of 80 500 000 copies/mL at Day 1, initiated antiretroviral therapy (ART) at Day 50, and had a viral load of <200 copies/mL by Day 166. Subsequently, Participant H was non-adherent to ART and had detectable N74D and viremia through Day 440 (final visit) but no emergence of additional CA substitutions (Supplementary Table 3). Participants I and J started ART early (Days 5 and 10, respectively) and had no resistance emergence, although the CA T107T/A polymorphism was detected in Participant J at Day 29. Participant K received F/TAF and developed M184M/V by Day 41, with a viral load of 36 200 000 copies/mL. Participant K initially declined ART but subsequently started DTG/3TC/TDF at Day 41 and achieved virologic suppression by Day 101 (Supplementary Table 3). Non-study drug resistance was detected in 20 of the 60 P1 participants tested for resistance. The NNRTI resistance mutations K103N, V106M, E138A/Q, Y188L, G190A, H221Y, and P225H, and the integrase strand transfer inhibitor (INSTI) resistance mutations T66I and E92E/G, were among the mutations detected (Table 3; Supplementary Table 4) and likely represent transmitted drug resistance in this population. The LEN resistance-associated mutation K70R was also detected in one South African participant with a subtype C virus and no exposure to LEN. This is likely a rare polymorphism.

**Table 3.** Resistance Analysis of Unrecognized Baseline Infections in PURPOSE 1

Participant	Group	HIV Subtype	Timepoint of Resistance Testing	Viral Load, Copies/mL	LEN-R	NRTI-R^a^	NNRTI-R	INSTI-R
G	LEN	A1	Day 1	105 000 000	–	–	–	–
			Day 9	227 000	N74N/D	–	–	E92E/G
			Day 176	271	N74D	–	–	–
H	LEN	D	Day 1	80 500 000	–	–	–	–
			Day 271	127 000	N74D	–	–	–
			Day 440	3340	N74D	–	–	–
I	LEN	C	Day 1	454 000	–	–	–	–
			Day 8	26 500	–	–	–	–
J	LEN	C	Day 1	129 000	–	–	–	–
			Day 29	353	T107T/A^b^	–	–	–
K	F/TAF	C	Day 1	36 200 000	–	–	–	–
			Day 41	22 900	–	M184M/V	–	–
L	F/TDF	A/D	Day 1	47 500	–	–	–	–
			Day 96	67 100	–	–	H221H/Y	–
M	F/TDF	C	Day 1	512 000	–	–	–	–
			Day 15	1130	–	AF	AF	AF

Abbreviations: –, no resistance mutations detected; AF, assay failure; F/TAF, emtricitabine/tenofovir alafenamide; F/TDF, emtricitabine/tenofovir disoproxil fumarate; INSTI, integrase strand transfer inhibitor; LEN, lenacapavir; NNRTI, non-nucleoside reverse transcriptase inhibitor; NRTI, nucleoside reverse transcriptase inhibitor; -R, resistance.

^a^F/TAF and F/TDF are NRTIs.

^b^T107A is currently considered a polymorphism based on lack of phenotypic resistance observed in vitro and in vivo.

### PURPOSE 2

Of the 3265 participants in the P2 EAP, 11 acquired HIV-1 after Day 1: nine in the F/TDF group and two in the LEN group (Table 1; Figure 3) [9].

In the LEN group, Participants D and E acquired HIV-1 at Weeks 13 and 26, respectively, and the N74D CA substitution emerged in both (Table 2), as described previously [9]. Viral load was 699 000 and 14 100 copies/mL at the time of HIV-1 detection for Participants D and E, respectively. Standard viral load and SCA testing were performed retrospectively, and while standard viral load testing was negative, SCA testing found that Participant D had a viral load of 4.81 copies/mL (95% CI: 1.67–13.88) at Week 8, but no resistance testing could be performed. All other samples tested by viral load and SCA for both participants were negative for HIV-1 RNA.

In the F/TDF group, one participant (F) developed the M184V substitution. Participant F had a negative HIV-1 test at Week 26 but a positive central laboratory HIV-1 test at Week 39. Retrospective NAAT was positive at Week 26, with no resistance detected; however, the M184V substitution had emerged by Week 39. Adherence was low at Weeks 8, 13, and 26 (Figure 2; Supplementary Table 2). ART was initiated 2 weeks post-diagnosis, with viral suppression (<200 copies/mL) achieved within 2 weeks.

Additionally, six participants from P2 acquired HIV-1 before drug administration at Day 1 and were analyzed for resistance (LEN [*n* = 4], F/TDF [*n* = 2]; Figure 3, Table 4). Resistance data were available for three of four participants who received LEN (Participants N, O, and P), with no resistance mutations observed at the earliest timepoint available for resistance testing (Day 1 for Participants N and P; Day 21 for Participant O). Participant N had a Day 1 viral load of 67 300 000 copies/mL, with emergence of N74D at Day 111. Participant N started DTG/3TC/TDF at Day 110 and had HIV-1 RNA <50 copies/mL by Day 184 (Supplementary Table 3). Participant O had a viral load of 31 copies/mL at Day 1; therefore, no resistance testing was performed until Day 21 (viral load of 189 000 copies/mL), with no resistance detected. At Day 77, the viral load was 78 600 copies/mL and N74D had emerged. Participant O initiated ART at Day 77 and had a viral load of 166 copies/mL at Day 119 (last visit). Participant P had no resistance present at Day 1 (viral load of 77 900 000 copies/mL) or Day 27 (final timepoint tested). Participant R, who received F/TDF, had a Day 1 viral load of 90 600 000 copies/mL and no resistance present. At Day 70, Participant R had a NAAT result of 93 000 copies/mL and M184M/V had emerged. Participant R initiated DTG/3TC/TDF at Day 117. At Day 209, their viral load was <20 copies/mL.

**Table 4.** Resistance Analysis of Unrecognized Baseline Infections in PURPOSE 2

Participant	Group	HIV Subtype	Timepoint of Resistance Testing	Viral Load, Copies/mL	LEN-R	NRTI-R^a^	NNRTI-R	INSTI-R
N	LEN	C	Day 1	67 300 000	–	–	–	–
			Day 111	65 400	N74D	–	–	–
O	LEN	C	Day 21^b^	189 000	–	–	–	–
			Day 77	78 600	N74D	–	–	–
P	LEN	C	Day 1	77 900 000	–	–	–	–
			Day 27	618	–	–	–	–
Q	LEN	Unknown	Day 1	452	AF	AF	AF	AF
			Day 16^c^	<20	N/A	N/A	N/A	N/A
R	F/TDF	F1	Day 1	90 600 000	–	–	–	–
			Day 70	93 000	–	M184M/V	–	–
S	F/TDF	B	Day 1	207	–	AF	AF	AF
			Day 23	528	–	AF	AF	AF

Abbreviations: –, no resistance mutations detected; AF, assay failure; F/TAF, emtricitabine/tenofovir alafenamide; F/TDF, emtricitabine/tenofovir disoproxil fumarate; INSTI, integrase strand transfer inhibitor; LEN, lenacapavir; N/A, not available; NNRTI, non-nucleoside reverse transcriptase inhibitor; NRTI, nucleoside reverse transcriptase inhibitor; -R, resistance; VL, viral load.

^a^F/TAF and F/TDF are NRTIs.

^b^Participant O had a VL of 31 copies/mL at Day 1 and testing for resistance was not attempted.

^c^No sample with VL >200 copies/mL to test.

Non-study drug resistance was detected in 2 of 17 participants from P2 and included the NNRTI resistance-associated substitution K103N (*n* = 2) and the protease inhibitor resistance-associated substitution V82L (*n* = 1; Table 2, Supplementary Table 4).

The LANL database analysis demonstrated that the N74 position was highly conserved in CA (Supplementary Table 5). Of the 8170 subtype C CA sequences, only one contained N74D, corresponding to a prevalence of 0.01% in this subtype. The N74D substitution occurred at a prevalence of 0.3% in subtype D and was not observed in subtypes A1 or AD.

## DISCUSSION

In the previous findings from P1 and P2, LEN demonstrated high efficacy, with a significantly smaller number of HIV-1 acquisitions compared with both bHIV and F/TDF [8, 9]. In the LEN groups, no participants acquired HIV-1 in P1 and only two acquired HIV-1 in P2 [8, 9]. Both participants from P2 who acquired HIV-1 during the study had the LEN resistance-associated substitution N74D present when HIV-1 was detected, with LEN plasma concentrations described as within the normal range [9]. Analysis of HIV subtype sequences in the LANL database showed that the N74 residue was highly conserved and N74D was found at very low levels, suggesting that transmitted N74D did not contribute to HIV acquisition while on LEN. Therefore, HIV was likely acquired while on study drug and the emergence of the N74D substitution was likely due to LEN monotherapy.

In studies of other PrEP agents, rapid point-of-care Ag/Ab testing has led to delays in HIV diagnosis due to suppressed antibody responses while on PrEP [25–27]. These delays have contributed to the emergence of resistance, primarily seen with long-acting agents such as cabotegravir, which may have been prevented by earlier HIV-1 detection. However, for the two participants who acquired HIV in the LEN group in P2, this explanation is unlikely. Both HIV acquisitions in the LEN group were detected using standard central laboratory serologic testing (fourth-generation Ag/Ab test). Retrospective testing of stored specimens demonstrated that neither participant had evidence of viremia preceding serologic diagnosis using standard quantitative NAAT. Participant D, who had a positive HIV-1 test at Week 13, was determined by retrospective SCA testing to have acquired HIV at the Week 8 visit. Since the participant was negative for HIV-1 at the Week 4 visit, the low viral load at Week 8 only 5 weeks before the Week 13 positive HIV-1 test suggests that HIV had been recently acquired and that the SCA finding was not due to inhibition attributable to LEN PrEP. Therefore, the diagnostic patterns observed in these participants suggest these acquisitions occurred shortly before diagnosis and that a more sensitive HIV-1 test from an earlier timepoint would not have expedited diagnosis or prevented resistance emergence.

Emergence of resistance with LEN monotherapy has been previously documented. In vitro resistance selection studies with LEN demonstrated initial emergence of the N74D substitution in both cell lines and primary cells, followed by the Q67H/N74D variant [7]. The Phase 2/3 CAPELLA study (NCT04150068) evaluated LEN in combination with an OBR in HTE participants with limited treatment options available [15, 16]. At Week 104, LEN resistance emerged in 14 participants receiving functional LEN monotherapy due to either poor adherence or not having fully active agents in their OBR [17]. Of note, the study reported one participant who had the N74D substitution emerge alone and later resuppressed without a change in regimen [17]. Indeed, despite emergence of resistance to LEN in these 14 CAPELLA participants, all maintained LEN treatment, and seven participants were able to resuppress through the end of study [17]. This HTE population allowed for the unique opportunity to evaluate resuppression in the presence of LEN resistance, which would generally not be done with daily oral treatments [17]. While emergence of resistance was rare in P1 and P2, more work is needed to determine whether a future LEN-containing HIV treatment regimen could be used in the setting of an isolated N74D mutation.

The N74D substitution was also observed in four of eight participants with unrecognized HIV at baseline in P1 and P2. Emergence of resistance in individuals who acquire HIV before initiating PrEP is well documented for all PrEP agents [28–31]. In P1 and P2, resistance was often associated with very high viral loads or extended delay of ART initiation. Of the four participants with unrecognized HIV at baseline, three had high viral loads at Day 1, suggesting that high levels of HIV replication at the time of LEN exposure may have resulted in a greater opportunity for resistance development. While the fourth participant did not present with a high viral load, it is suspected that a delay in their ART initiation led to functional LEN monotherapy, which allowed for resistance to develop. Indeed, delays in ART initiation were observed in several other PURPOSE participants with unrecognized HIV at baseline who received daily oral PrEP and later developed other resistance mutations, highlighting the importance of rapid ART initiation in participants who acquire HIV after receiving PrEP. In addition to the four participants who developed N74D, one other participant with unrecognized HIV at baseline developed the T107T/A substitution while on LEN. The T107A substitution is a documented polymorphism and no phenotypic resistance has been observed in vitro or in vivo [17, 32]. Further work is being conducted to evaluate the additional role it could be playing as a possible secondary or compensatory mutation.

The N74D substitution has been shown to have reduced susceptibility to LEN compared with wild type (WT) in vitro (14–22 fold) [11]. In vivo, the CAPELLA study reported two participants who developed N74D after the emergence of other CA resistance-associated substitutions [17]. Phenotypic testing of these samples showed high levels of resistance, which increased with the N74D substitution (16- to >869-fold changes in LEN susceptibility compared with WT) [17]. Phenotyping of the clinical isolates in the PURPOSE trials was not attempted; no susceptibility data were available. However, further emergence of CA resistance in addition to N74D was not observed, even in cases of unrecognized HIV at baseline where N74D was detected early in the study.

Study drug resistance was rare in the F/TAF and F/TDF groups and often associated with low or fluctuating adherence, similar to previous studies [28, 30, 31, 33, 34]. However, one participant in P1 developed both emtricitabine and TFV resistance despite a high level of adherence up until HIV acquisition. In this case, it is likely that resistance occurred by transmitted drug resistance, which is also supported by the coacquisition of an NNRTI resistance mutation. Two participants with unrecognized HIV at baseline (one in P1 receiving F/TAF; one in P2 receiving F/TDF) had emergence of the M184M/V mutation. However, as adherence data were not collected, the impact of adherence is unknown. Nevertheless, these findings highlight the importance of PrEP adherence in preventing HIV acquisition, while also demonstrating the importance of rapid ART start in preventing the development of resistance.

It is important to note some limitations of the current work. First, the small number of failures, especially in the LEN group, limits the interpretation of overall resistance conclusions for LEN for PrEP. Also, while most of the participants who acquired HIV-1 in the PURPOSE trials were analyzed for resistance, genotype data were not available for four participants in P1. In addition, the two participants who acquired HIV in the LEN group during the study in P2 were lost to follow-up, and therefore long-term data were unavailable for these participants. Despite these limitations, the collation of data across two clinical trials in diverse populations adds strength to the generalizability of the findings.

In summary, although LEN was highly efficacious for HIV prevention in P1 and P2, emergent LEN resistance was observed in the rare incident HIV cases that occurred during LEN use, as well as among some participants with unrecognized HIV at baseline who began LEN. Importantly, in this limited number of cases, LEN use did not appear to delay HIV diagnosis and first-line INSTI-based ART retained full antiviral activity against the identified HIV isolates. Although further work is needed to completely understand the diagnostic patterns of HIV acquisition in the context of LEN use and the implications of LEN resistance development when HIV acquisition occurs, it seems likely that the adherence, persistence and efficacy advantages of LEN will outweigh the risk of resistance development.

## Supplementary Material

jiaf533_Supplementary_Data

## Supplementary Data

Supplementary materials are available at *The Journal of Infectious Diseases* online (http://jid.oxfordjournals.org/). Supplementary materials consist of data provided by the author that are published to benefit the reader. The posted materials are not copyedited. The contents of all supplementary data are the sole responsibility of the authors. Questions or messages regarding errors should be addressed to the author.
