# Supplementary material for: Resistance Analyses of Lenacapavir, Emtricitabine/Tenofovir Alafenamide and Emtricitabine/Tenofovir Disoproxil Fumarate in the PURPOSE 1 and 2 Studies
Source: J Infect Dis. 2025 Oct 24;233(1):e203–11. doi: 10.1093/infdis/jiaf533 (PMC12811884; doi:10.1093/infdis/jiaf533)
Supplement: jiaf533_Supplementary_Data [file jiaf533_supplementary_data.zip › Supplementary Figure 1.docx]

**
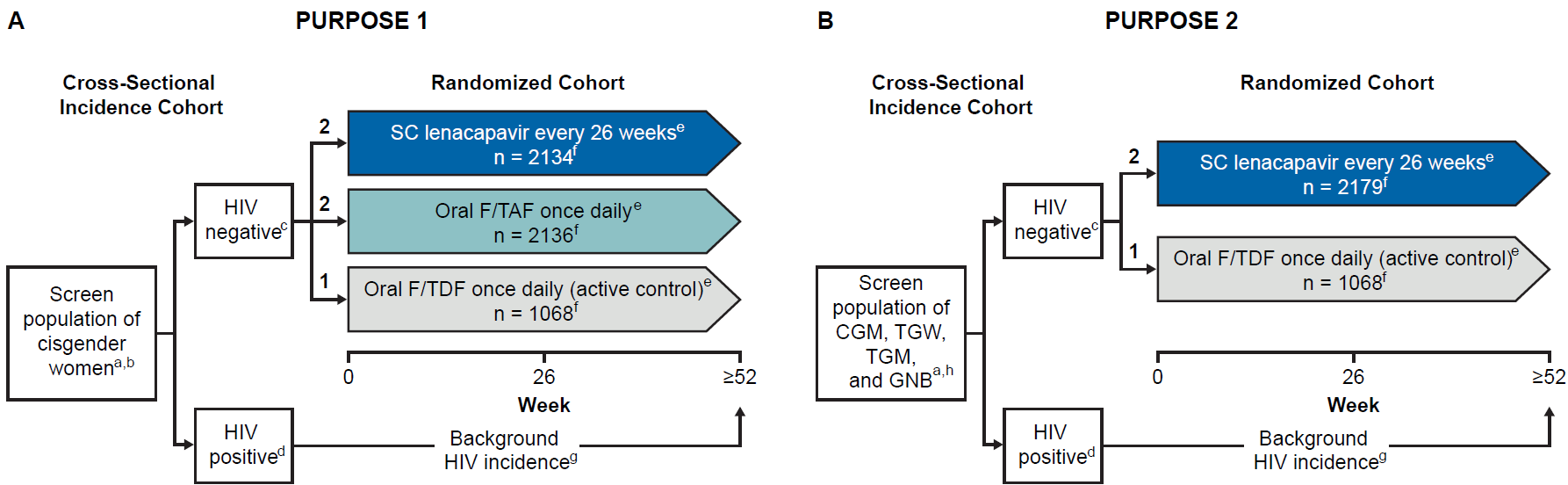
Supplementary Figure 1.** PURPOSE 1 (A) and PURPOSE 2 (B) study designs. ^a^Not receiving PrEP and without HIV testing in past 3 months. ^b^The first participant was screened in August 2021, the 50th-percentile participant was randomized in May 2023 and the last participant was randomized in September 2023. ^c^Participants also met eligibility criteria (including body weight ≥35 kg, eGFR ≥60 mL/min, not pregnant). ^d^Recency assay data were used to estimate background HIV incidence (persons testing HIV positive were linked to HIV care). ^e^Plus the alternative SC/oral placebo. ^f^n numbers represent the full analysis set for efficacy analyses. ^g^Background HIV incidence was the incidence expected in the absence of PrEP and was analogous to a placebo group. ^h^The first participant was screened in June 2021, the 50th-percentile participant was randomized in August 2023 and the last participant was randomized in December 2023.
CGM, cisgender men; eGFR, estimated glomerular filtration rate; F/TAF, emtricitabine/tenofovir alafenamide; F/TDF, emtricitabine/tenofovir disoproxil fumarate; GNB, gender nonbinary; PrEP, preexposure prophylaxis; SC, subcutaneous; TGM, transgender men; TGW, transgender women.
