# Supplementary material for: Resistance Analyses of Lenacapavir, Emtricitabine/Tenofovir Alafenamide and Emtricitabine/Tenofovir Disoproxil Fumarate in the PURPOSE 1 and 2 Studies
Source: J Infect Dis. 2025 Oct 24;233(1):e203–11. doi: 10.1093/infdis/jiaf533 (PMC12811884; doi:10.1093/infdis/jiaf533)
Supplement: jiaf533_Supplementary_Data [file jiaf533_supplementary_data.zip › Supplementary Table 1.docx]

**Supplementary Table 1. Major Primary Resistance Mutations Evaluated and Their Associations With ART Medications**

| Resistance Type | Mutations |
| --- | --- |
| NRTI | M41L, K65R/E/N, D67N, T69D/N, T69Del/Ins, K70E/R, L74I/ V, V75I, F77L, Y115F, F116Y, Q151M, M184I/V, L210W, T215F/Y, K219E/N/Q/R. |
| NNRTI | L100I, K101E/H/P, K103N/S, V106A/M, V108I, E138A/G/K/ Q/R, V179L, Y181C/I/V, Y188C/H/L, G190A/Q/S, H221Y, P225H, F227C, M230I/L. |
| PI | D30N, V32I, M46I/L, I47A/V, G48V, I50L/V, I54L/M/V, Q58E, T74P, L76V, V82A/F/L/S/T, N83D, I84V, N88S, L90M. |
| INSTI | T66A/I/K, E92G/Q/V, G118R, F121C/Y, G140R, Y143C/H/R, S147G, Q148H/K/R, N155H/S, R263K. |
| CAI | L56I, M66I, Q67H/K/N, K70H/N/S/R, N74D/S, A105S/T, T107A/C/N/S |

Abbreviations: CAI, capsid inhibitor; INSTI, integrase strand transfer inhibitor; NNRTI, non-nucleoside reverse transcriptase inhibitor; NRTI, nucleoside reverse transcriptase inhibitor; PI, protease inhibitor.
