# Supplementary material for: Resistance Analyses of Lenacapavir, Emtricitabine/Tenofovir Alafenamide and Emtricitabine/Tenofovir Disoproxil Fumarate in the PURPOSE 1 and 2 Studies
Source: J Infect Dis. 2025 Oct 24;233(1):e203–11. doi: 10.1093/infdis/jiaf533 (PMC12811884; doi:10.1093/infdis/jiaf533)
Supplement: jiaf533_Supplementary_Data [file jiaf533_supplementary_data.zip › Supplementary Table 2.docx]

**Supplementary Table 2. Adherence to Treatment in PURPOSE 1 and PURPOSE 2 Participants Who Developed Resistance**

| Participant | Group | HIV Diagnosis Analysis Visit, Week | Analysis Visit, Week | TFV-DP, fmol/Punch | FTC-TP, fmol/Punch |
| --- | --- | --- | --- | --- | --- |
| PURPOSE 1 |  |  |  |  |  |
| A | F/TAF | 26 | 4 | 176.69 | BLQ |
|  |  |  | 8 | 729.16 | 3.33 |
|  |  |  | 13 | 991.24 | 4.31 |
|  |  |  | 26 | 404.83^a^ | 1.47 |
| B | F/TAF | 8 | 4 | 1315.58 | 5.80 |
|  |  |  | 8 | 1779.20^a^ | 5.83 |
| C | F/TDF | 26 | 4 | 133.11 | 0.15 |
|  |  |  | 8 | 366.37 | 0.35 |
|  |  |  | 13 | 224.28 | BLQ |
|  |  |  | 26 | 65.96^a^ | BLQ |
| PURPOSE 2 |  |  |  |  |  |
| F | F/TDF | 26 | 8 | BLQ | BLQ |
|  |  |  | 13 | 276.20 | BLQ |
|  |  |  | 26 | 86.66 | BLQ |

Abbreviations: BLQ, below the limit of quantification; F/TAF, emtricitabine/tenofovir alafenamide; FTC-TP, emtricitabine triphosphate; F/TDF, emtricitabine/tenofovir disoproxil fumarate; TFV-DP, tenofovir diphosphate.

^a^DBS concentration on HIV-1 diagnosis date for cases.
