# Supplementary material for: Resistance Analyses of Lenacapavir, Emtricitabine/Tenofovir Alafenamide and Emtricitabine/Tenofovir Disoproxil Fumarate in the PURPOSE 1 and 2 Studies
Source: J Infect Dis. 2025 Oct 24;233(1):e203–11. doi: 10.1093/infdis/jiaf533 (PMC12811884; doi:10.1093/infdis/jiaf533)
Supplement: jiaf533_Supplementary_Data [file jiaf533_supplementary_data.zip › Supplementary Table 3.docx]

**Supplementary Table 3. Details of PURPOSE 1 and PURPOSE 2 Participants With Resistance Testing**

| Participant | Day  (Analysis Visit, Weeks) | Viral Load | Resistance Testing Performed | Study Drug Resistance Detected | ART Regimen |
| --- | --- | --- | --- | --- | --- |
| PURPOSE 1 |  |  |  |  |  |
| A^a^ | 1 (BL) | No HIV-1 RNA detected |  |  |  |
|  | 96 (13) | No HIV-1 RNA detected |  |  |  |
|  | 189 (26) | 4500 | R | M184M/I |  |
|  | 293 (39) | 752 |  |  |  |
|  | 309 (39) | 42 |  |  |  |
| B | 1 (BL) | No HIV-1 RNA detected |  |  |  |
|  | 29 (4) | No HIV-1 RNA detected |  |  |  |
|  | 57 (8) | 6890 | R | K65K/R, M184M/I |  |
|  | 92 (13) | 1510 | R | M184I |  |
|  | 103 (13) |  |  |  | DTG+3TC+TDF |
|  | 115 (13) | <20 copies/mL HIV-1 RNA detected |  |  |  |
| C | 1 (BL) | No HIV-1 RNA detected |  |  |  |
|  | 97 (13) | No HIV-1 RNA detected |  |  |  |
|  | 176 (26) | 38 200 | R | None |  |
|  | 268 (39) | 108 000 | R | M184M/I/V |  |
|  | 282 (39) |  |  |  | DTG+3TC+TDF |
|  | 310 (39) | 112 000 |  |  |  |
|  | 365 (52) | 165 000 |  |  |  |
|  | 463 (65) | <20 copies/mL HIV-1 RNA detected |  |  |  |
| G | –24 (Scr) | No HIV-1 RNA detected |  |  |  |
|  | 1 (BL) | 105 000 000 | R | None |  |
|  | 9 (4) | 227 000 | R | N74N/D |  |
|  | 141 (26) |  |  |  | DTG+3TC+TDF |
|  | 176 (26) | 271 | R | N74D |  |
|  | 269 (39) | 168 |  |  |  |
|  | 361 (52) | 90 |  |  |  |
| H | –15 (Scr) | No HIV-1 RNA detected |  |  |  |
|  | 1 (BL) | 80 500 000 | R | None |  |
|  | 50 (8) |  |  |  | ART (NOS) |
|  | 75 (13) | 198 |  |  |  |
|  | 166 (26) | 119 |  |  |  |
|  | 271 (39) | 127 000 | R | N74D |  |
|  | 347 (52) | 12 300 | R | N74D |  |
|  | 440 (65) | 3340 | R | N74D |  |
| I | –14 (Scr) | No HIV-1 RNA detected |  |  |  |
|  | 1 (BL) | 4 540 000 | R | None |  |
|  | 6 (4) |  |  |  | DTG+3TC+TDF |
|  | 8 (4) | 26 500 | R | None |  |
|  | 29 (4) | 195 |  |  |  |
|  | 91 (13) | <20 copies/mL HIV-1 RNA detected |  |  |  |
| J | –25 (Scr) | No HIV-1 RNA detected |  |  |  |
|  | 1 (BL) | 129 000 | R | None |  |
|  | 15 (4) | 269 000 |  |  |  |
|  | 24 (4) |  |  |  | DTG+3TC+TDF |
|  | 29 (4) | 353 | R | T107T/A^b^ |  |
|  | 95 (13) | No HIV-1 RNA detected |  |  |  |
| K | –15 (Scr) | No HIV-1 RNA detected |  |  |  |
|  | 1 (BL) | 36 200 000 | R | None |  |
|  | 9 (4) | 191 000 |  |  |  |
|  | 41 (4) | 22 900 | R | M184M/V |  |
|  | 52 (8) |  |  |  | DTG+3TC+TDF |
|  | 101 (13) | No HIV-1 RNA detected |  |  |  |
| L | –17 (Scr) | No HIV-1 RNA detected |  |  |  |
|  | 1 (BL) | 47 500 | R | None |  |
|  | 7 (4) | 59 000 |  |  |  |
|  | 13 (4) | 187 000 |  |  |  |
|  | 15 (4) |  |  |  | DTG+3TC+TDF |
|  | 96 (13) | 67 100 | R | None |  |
|  | 291 (39) | <20 copies/mL HIV-1 RNA detected |  |  |  |
| M | –15 (Scr) | No HIV-1 RNA detected |  |  |  |
|  | 1 (BL) | 512 000 | R | None |  |
|  | 8 (4) |  |  |  | DTG+3TC+TDF |
|  | 15 (4) | 1130 | R | AF |  |
|  | 43 (8) | 62 |  |  |  |
|  | 49 (8) | 43 |  |  |  |
|  | 100 (13) | No HIV-1 RNA detected |  |  |  |
| PURPOSE 2 |  |  |  |  |  |
| D^a^ | (Scr) (-11) | No HIV-1 RNA detected |  |  |  |
|  | 1 (BL) | No HIV-1 RNA detected |  |  |  |
|  | 28 (4) | No HIV-1 RNA detected |  |  |  |
|  | 56 (8) | No HIV-1 RNA detected |  |  |  |
|  | 93 (13) | 699 000 | R | N74D |  |
| E^a^ | (Scr) (-24) | No HIV-1 RNA detected |  |  |  |
|  | 1(BL) | No HIV-1 RNA detected |  |  |  |
|  | 92 (13) | No HIV-1 RNA detected |  |  |  |
|  | 185 (26) | 14 100 | R | N74D |  |
| F | (Scr) (-12) | No HIV-1 RNA detected |  |  |  |
|  | 1 (BL) | No HIV-1 RNA detected |  |  |  |
|  | 94 (13) | No HIV-1 RNA detected |  |  |  |
|  | 183 (26) | 209 000 | R | None |  |
|  | 283 (39) | 99 700 | R | M184V |  |
|  | 297 (39) |  |  |  | DTG+3TC+TDF |
|  | 311 (39) | 185 |  |  |  |
| N | –28 (Scr) | No HIV-1 RNA detected |  |  |  |
|  | 1 (BL) | 67 300 000 | R | None |  |
|  | 20 (4) | 4340 |  |  |  |
|  | 111 (13) | 65 400 | R | N74D |  |
|  | 112 (13) |  |  |  | DTG+3TC+TDF |
|  | 184 (26) | <20 copies/mL HIV-1 RNA detected |  |  |  |
|  | 258 (39) | No HIV-1 RNA detected |  |  |  |
| O | –21 (Scr) | No HIV-1 RNA detected |  |  |  |
|  | 1 (BL) | 31 |  |  |  |
|  | 21 (4) | 189 000 | R | None |  |
|  | 29 (4) | 944 000 |  |  |  |
|  | 64 (8) |  |  |  | DTG+3TC+TDF |
|  | 77 (13) | 78 600 | R | N74D |  |
|  | 119 (13) | 166 |  |  |  |
| P | –34 (Scr) | No HIV-1 RNA detected |  |  |  |
|  | 1 (BL) | 77 900 000 | R | None |  |
|  | 27 (4) | 618 | R | None | DTG+3TC+TDF |
|  | 62 (8) | 36 |  |  |  |
|  | 126 (13) | 27 |  |  |  |
| Q | –28 (Scr) | No HIV-1 RNA detected |  |  |  |
|  | 1 (BL) | 452 | R | AF |  |
|  | 16 (4) | <20 copies/mL HIV-1 RNA detected |  |  |  |
|  | 32 (4) | No HIV-1 RNA detected |  |  | DTG+3TC |
|  | 71 (8) | No HIV-1 RNA detected |  |  |  |
|  | 122 (13) | No HIV-1 RNA detected |  |  |  |
| R | –29 (Scr) | No HIV-1 RNA detected |  |  |  |
|  | 1 (BL) | 90 600 000 | R | None |  |
|  | 20 (4) | 104 000 |  |  |  |
|  | 70 (8) | 93 000 | R | M184M/V |  |
|  | 100 (13) |  |  |  | DTG+3TC+TDF |
|  | 117 (13) | 106 000 | R | None |  |
|  | 209 (26) | <20 copies/mL HIV-1 RNA detected |  |  |  |
| S^a^ | –21 (Scr) | No HIV-1 RNA detected |  |  |  |
|  | 1 (BL) | 207 | R | AF |  |
|  | 8 (4) | 1710 | R | AF |  |
|  | 23 (4) | 528 | R | AF |  |
|  | 56 (8) | No HIV-1 RNA detected |  |  |  |

Abbreviations: 3TC, lamivudine; AF, assay failure; ART, antiretroviral therapy; BL, baseline;; DTG, dolutegravir; ND, not determined; NOS, not otherwise specified; R, resistance detected; TDF, tenofovir disoproxil fumarate.

All participants received their first injection at Day 1.

^a^No information on ART regimen was available for these participants.

^b^T107A is currently considered a polymorphism based on lack of phenotypic resistance observed in vitro and in vivo.
