# Supplementary material for: Resistance Analyses of Lenacapavir, Emtricitabine/Tenofovir Alafenamide and Emtricitabine/Tenofovir Disoproxil Fumarate in the PURPOSE 1 and 2 Studies
Source: J Infect Dis. 2025 Oct 24;233(1):e203–11. doi: 10.1093/infdis/jiaf533 (PMC12811884; doi:10.1093/infdis/jiaf533)
Supplement: jiaf533_Supplementary_Data [file jiaf533_supplementary_data.zip › Supplementary Table 4.docx]

**Supplementary Table 4. Transmitted Drug Resistance in PURPOSE 1 and PURPOSE 2 Participants**

| Participant | Group | HIV Subtype | Study Week of Infection | Viral Load, copies/mL | TFV-DP, fmol/punch (Adherence Level)^a^ | CAI-R | NRTI-R | NNRTI-R | PI-R | INSTI-R |
| --- | --- | --- | --- | --- | --- | --- | --- | --- | --- | --- |
| PURPOSE 1 | | | | | | | | | | |
| T | F/TAF | C | 78 | 539 | BLQ (low) | AF | - | E138A | - | - |
| U | F/TAF | C | 52 | 17 700 000 | 87.94 (low) | - | - | E138A | - | - |
| V | F/TAF | A | 52 | 95 700 | BLQ (low) | - | - | E138A | - | - |
| A | F/TAF | C | 26 | 4500 | 404.83 (low) | - | M184M/I | - | - | - |
| W | F/TAF | C | 52 | 2 950 000 | BLQ (low) | - | - | E138Q | - | - |
| X | F/TAF | A1 | 26 | 258 000 | BLQ (low) | - | - | K103K/N | - | - |
| Y | F/TAF | C | 39 | 33 900 | BLQ (low) | K70R | - | - | - | - |
| Z | F/TAF | C | 26 | 3 620 000 | BLQ (low) | - | - | K103N | - | - |
| AA | F/TAF | C | 26 | 2 270 000 | BLQ (low) | - | - | - | - | - |
| B | F/TAF | C | 8 | 6890 | 1779.20 (high) | - | K65K/R, M184M/I | Y188L | - | - |
|  |  |  | 13 | 1510 | ND | - | M184I | Y188L | - | - |
| BB | F/TAF | C | 13 | 448 | ND | - | - | E138A | - | - |
| CC | F/TAF | A1 | 39 | 2 320 000 | 129.30 (low) | - | - | G190A | - | T66I |
| DD | F/TAF | C | 13 | 617 000 | 63.76 (low) | - | - | K103N, V106M | - | - |
| EE | F/TDF | C | 65 | 21 600 | ND | - | - | K103N, P225H | - | - |
| C | F/TDF | C | 26 | 38 200 | 65.96 (low) | - | - | K103N | - | - |
|  |  |  | 39 | 108 000 | ND | - | M184M/I/V | K103N | - | - |
| FF | F/TDF | C | 39 | 14 200 | BLQ (low) | - | - | K103N | - | - |
| GG | F/TDF | C | 26 | 128 000 | 220.76 (low) | - | - | K103N | - | - |
| HH | F/TDF | C | 39 | 13 600 | BLQ (low) | - | - | Y181C | - | - |
| II | F/TDF | C | 39 | 1 080 000 | 142.71 (low) | - | - | K103N | - | - |
| JJ | F/TDF | C | 26 | 9260 | BLQ (low) | - | - | E138A | - | - |
| PURPOSE 2 | | | | | | | | | | |
| D | LEN | C | 13 | 699 000 | ND | N74D | - | K103N | - | - |
| E | LEN | C | 26 | 14 100 | ND | N74D | - | - | - | - |
| F | F/TDF | C | 26 | 209 000 | 86.66 (low) | - | - | - | V82L | - |
|  |  |  | 39 | 99 700 | ND | - | M184V | - | V82L | - |
| KK | F/TDF | B | 26 | 3 180 000 | BLQ (low) | - | - | K103N | - | - |

Abbreviations: -, no resistance mutations detected; AF, assay failure; BLQ, below the limit of quantification; CAI, capsid inhibitor;; F/TAF, emtricitabine/tenofovir alafenamide; F/TDF, emtricitabine/tenofovir disoproxil fumarate; TFV-DP, tenofovir diphosphate; INSTI, integrase strand transfer inhibitor; LEN, lenacapavir; ND, not determined; NNRTI, non-nucleoside reverse transcriptase inhibitor; NRTI, nucleoside reverse transcriptase inhibitor; PI, protease inhibitor; -R, resistance.

^a^In dried blood spots.
