# Supplementary material for: Resistance Analyses of Lenacapavir, Emtricitabine/Tenofovir Alafenamide and Emtricitabine/Tenofovir Disoproxil Fumarate in the PURPOSE 1 and 2 Studies
Source: J Infect Dis. 2025 Oct 24;233(1):e203–11. doi: 10.1093/infdis/jiaf533 (PMC12811884; doi:10.1093/infdis/jiaf533)
Supplement: jiaf533_Supplementary_Data [file jiaf533_supplementary_data.zip › Supplementary Table 5.docx]

**Supplementary Table 5. N74 Mutations in Sequences From the LANL Database**

| HIV Subtype  **(N=12,075)** | Mutation | Count  **(N=12,075)** | Prevalence (%) |
| --- | --- | --- | --- |
| A1 (N=2946) | N | 2941 | 99.8 |
|  | S | 2 | 0.07 |
|  | K | 1 | 0.03 |
|  | H | 1 | 0.03 |
|  | T | 1 | 0.03 |
| AD (N=28) | N | 28 | 100.0 |
| C (N=8170) | N | 8161 | 99.9 |
|  | K | 3 | 0.04 |
|  | T | 2 | 0.02 |
|  | Y | 2 | 0.02 |
|  | D | 1 | 0.01 |
|  | S | 1 | 0.01 |
| D (N=931) | N | 924 | 99.3 |
|  | D | 3 | 0.3 |
|  | E | 1 | 0.1 |
|  | F | 1 | 0.1 |
|  | I | 1 | 0.1 |
|  | K | 1 | 0.1 |

Abbreviation: LANL, HIV Los Alamos National Laboratory [35].
